# Medical and Philosophical Intelligence

**Published:** 1819-03

**Authors:** 


### MEDICAL AND PHILOSOPHICAL INTELLIGENCE.

WE shall devote a portion of the present Number of our Journal to a subject solely of local interest, which has been brought before the public by Mr. James Parkinson;* that of the propriety of establishing fever-wards in the different parishes of the metropolis.

The discussions that were commenced a short time since respecting the essential origin of the fever that has lately prevailed to a considerable extent in London and its suburbs, led to expectations that they would terminate in some decisive conclusions on that point; but they suddenly terminated with the public alarm that gave rise to them, without having effected what was so much to be desired.

Particular enquiries respecting the opinions of the generality of physicians, have shown that nearly all those who have had the most extensive opportunities for observation are disposed to consider it as essentially contagious; though its dissemination and effects are considerably influenced by local situations, and the habits and conditions of the patients of the exciting cause. Mr. Parkinson,

* In a pamphlet entitled "*Observations on the Necessity of Parochial Fever-Wards; with Remarks on the present extensive Spread of Fever;* by James Parkinson, Member of the Royal College of Surgeons."—8vo. pp. 20. Sherwood and Co. London.

who appears to have given much attention to the subject, is of this opinion. He thinks that the disease under consideration may be referred to a fever that occurred in the inhabitants of some houses in a court in Kingsland-road, in the parish of St. Leonard, Shoreditch; whence he traces it to its present epidemic state. Although many circumstances he details appear to show that local situation was essentially connected with its origin in some instances; yet other facts equally favour the opinion that it has, in other cases, been dispersed by contagion. But, whichever of these views be correct, the propriety of the measure urged by Mr. Parkinson with so much laudable zeal, is sufficiently obvious. The numerous scenes of wretchedness that he has witnessed in consequence of the ravages of the prevalent fever, where the victims have suffered every privation, in consequence of their inability to procure hired nurses, and the fears of their neighbours depriving them of gratuitous domestic aid, are such as cannot fail to excite the attention of all those who may become acquainted with them, and lead to exertions for the prevention of similar calamities in future.

"The first measure which should be adopted," says Mr. Parkinson, "is that of the establishment of fever-wards. It is, indeed, astonishing, in an age distinguished for its flow of charity and kindly consideration of the poor, that the warning voice of the Society for bettering the Condition of the Poor, and the anxious remonstrances of numerous respectable and benevolent individuals, have yet produced the establishment of only one place, furnished with accommodations for about seventy patients, for the reception of the pauper fever patients of London and its environs.

"An establishment with eighteen beds (six for males, and twelve for females,) would, it is expected, be sufficient for the paupers belonging to a parish whose population is from 40 to 50,000 persons; and the beds and the building might always be usefully employed as an infirmary, when the fever was subdued."

After having demonstrated the evil, and, indeed, the fatal consequences, of either keeping fever-patients in their lodging-houses, or of sending them to the poor-house, Mr. Parkinson briefly points out the advantages of placing them in a fever-ward.

"Immediately as a patient is affected with fever," he observes, "he is removed thither by a proper conveyance, his tatters immersed in water, and he is carefully washed and consigned to this ward; in which would be secured the means of ventilation and purification, the patient would gain every chance of his recovery, whilst the air of the ward itself would be preserved innoxious, and consequently the attendants would be exposed to much less danger. The patient, on his recovery, would be supplied with fresh clothing, and would return to society, no longer conveying about with him the poison of pestilence.

"Satisfied," continues the author, "that those to whom these lines are more particularly addressed are anxious to promote, on every occasion, the comfort of the poor and the welfare of society, not the slightest fear is entertained of all due attention being paid

4

to these suggestions, offered with combined earnestness and respect."

After pointing out the several advantages of minor importance that would ensue from the adoption of the measure he advises, Mr. Parkinson concludes with remarking, that, "should every parish provide fever-wards for their own poor, the necessity would still continue for the liberal support of the more general institution of this kind. Where else would be sent the servant, or other inmate, who ought not to have parochial relief claimed for them; but who, for the safety of the families with whom they reside, require immediate removal and separation from the healthy? Where could be sent the wretched and unprotected Irish poor, suffering here, in their own country, as it is called, all the privations and miseries of exile?"

---

There are few medical practitioners, we presume, who are unacquainted with the great and extensive influence which the contents of the Medical and Physical Journal had in the establishment of *vaccine inoculation;* and, for several years after that practice was first adopted, this Journal was the medium through which the most important information respecting it was originally conveyed to the public, as well as that in which much interesting discussion on it was pursued. A disposition to let nothing of consequence respecting it pass unrecorded in this work, leads us to notice *a Memoir on the present State of Vaccination,* by Mr. Thomas Brown, of Musselburgh, which has recently appeared in the fifty-eighth Number of the Edinburgh Medical and Surgical Journal, notwithstanding that it contains observations and remarks adverse to the interests of this subject.

Mr. Brown was one of the most early promoters of vaccination in Scotland, and continued to pursue the practice with unremitted zeal until the year 1808; before which time he had inoculated upwards of 1200 patients. At the time just referred to, some facts occurred to his observation which shook his confidence in the prophylactic powers of this measure against small-pox, and made him suspect that it would lead to the most serious evils, at some future period, in the consequences that would necessarily result from it should his fears prove well-founded. Mr. Brown then made public his observations and his sentiments respecting them; but the reliance placed on the measure they were intended to reprobate was so firm as to be proof against any temporary opposing force, however powerful and well directed.

The facts which extensive enquiries and attentive investigation have since then brought to the observation of Mr. Brown, and more especially those which have recently occurred to such an extent in various parts of Europe, have made such an impression on the mind of this intelligent surgeon, as to induce him again to press his opinions respecting them on the attention of medical practitioners.

After noticing the mild, and comparatively unimportant, character that small-pox had assumed when inoculation for that disease had been generally adopted, and some physiological phenomena, which he thinks would alone show the little power possessed by cow-pox to destroy essentially the influence of the virus of the former, Mr. Brown observes, that—

"Experience and experiment have shown that, upon having recourse to exposure to the epidemic small-pox, some years after having undergone vaccination, the individual is found to be influenced more or less by the contagion, and is liable to the disease of small-pox; and, if inoculation with small-pox is again tried at the interval of some years from vaccination, not only the perfect local phenomena of small-pox inoculation will be obtained, but also fever and eruption; and these two tests will now, in general, be found to exert their influence upon the constitution, exactly in conformity with the distance of the period from vaccination.

"In conformity with this observation, experience has shown that, for the first two or three years after the introduction of vaccination in Great Britain, scarcely a case of failure was heard of; but afterwards they have been gradually increasing in number and severity every successive year; and that the number of such cases of failure have been met with, in the respective situations, exactly in proportion to the early introduction of the Jennerian practice, the extent of population, and the proportion of the lower classes of the community.

"The accounts from all quarters of the world, wherever vaccination has been introduced, are assuming exactly the same aspect; and, at the present period, it cannot be disputed that the cases of failure are now increased to an alarming proportion; and, from a fair and impartial examination, it appears, where the small-pox contagion has access to operate upon vaccinated cases of upwards of six years' standing, and the contagion applied in a concentrated and lasting form, nearly the whole of such cases will yield to the influence of the small-pox contagion."—

—"In examining my own practice," Mr. Brown observes, in another part of his Memoir, "few or none escaped at the distance of six years after vaccination, that were placed in circumstances favourable for the operation of the epidemic; very few at four years; and the greatest number who resisted the contagion were either within four years, or not exposed to a concentrated and extensive application of the contagion."

We shall continue to transcribe the most important passages of the Memoir of Mr. Brown, without offering any comments on them; although, in justice to him, we must remark that he constantly refers to facts which he considers will prove the correctness of the assertions he has advanced.

"But it is not only in the number of failures that this subject becomes alarming, it is also in the severity of the disease; and at this period many of the instances of failure not only assume the most severe form of the distinct disease, but also have been confluent; and death has followed in a considerable number of cases."—

"It appears that neither this unfortunate imperfection of the vaccine process in giving security against small-pox contagion, nor of the variety in the phenomena which the process may undergo, can admit of any remedy. The vaccinations conducted by Dr. Jenner, by public institutions, by private practitioners, by ministers, midwives, or farriers, all have failed; whether the process has been conducted by one puncture, by two, or even by four; or whether the eventful test has been applied;* all have fallen short of the desired effect. The truth is, the only difference of the modes of conducting the vaccine process rests merely in extending or diminishing the period of security *a year or two*, and is but of little consequence exactly to ascertain.

"I am decidedly of opinion, there is no mode of giving the vaccine disease by inoculation, which can impart *perfect and permanent security* against small-pox contagion; and I apprehend it is impossible to give the disease in any other form, to obtain that effect. For, if any one will attentively examine the whole history of vaccination, from Dr. Jenner's publications downwards, he will find the proof of its powers to impart *permanent* security almost gratuitous."—"Indeed, Dr. Jenner's cases do not amount to thirty, where he stated such exemption had reached to the extent of thirty years, or for life; and certainly there is no improbability, or impossibility, in supposing such number either to be kept in a state of security, from continuing to exercise the office of milker, or to be those peculiarities of constitution not susceptible of small-pox; which number is certainly not too many to exist in the populous county of Gloucester."—

"With regard to the curious excuse contrived by Mr. Bryce to defeat those cases of small-pox which have occurred after his vaccinations, viz.—'that, although the vaccine phenomena are all present, and exist in their most perfect form, still the constitution is not affected by the vaccine disease, and is left exposed to the influence of small-pox contagion:' I really think, if we were to admit such a defence, it might be fairly concluded we have bid an eternal adieu to the exercise of our judgment. I shall leave it for you to determine, if it is not ridiculous and absurd in the extreme, after having described vaccination as so simple and so uniform, and to consist of certain phenomena which were invariably found to produce such an effect upon the constitution as to prevent any farther effects from a second vaccination; to resist small-pox inoculation, and exposure to the epidemic; and also, at first, nearly to annihilate the small-pox wherever the practice existed to any extent;—to come forward and maintain, merely because small-pox have succeeded to vaccination, that the process is intricate, uncertain, and difficult to conduct; that there are none of the phenomena to be depended upon as indicating the action of vaccination upon the system; that the resistance of the tests of re-vaccination, inoculation, or exposure to the epidemic, is not to be depended upon;

---

* Mr. Bryce's method of vaccinating with the test puncture.

that, although symptoms marking a constitutional derangement are present during the existence of the vaccine phenomena, still it is not owing to that process, *but to some diseased action of the constitution;* that the production of a small-pox pustule by inoculating those who had undergone vaccination some years before was of no consequence, even although attended with an areola and constitutional disorder, *because it is a matter of fact, and is either well known, or ought to be known, to every surgeon, that these can be produced at pleasure; and one of the gentlemen connected with the vaccine establishment in Edinburgh has kept up a supply of small-pox matter for inoculation, by successively inoculating his own arm.* The scab or crust which remains after the disease was completed, which was supposed to indicate the perfect disease, is, *after the failure has taken place,* found out to be too little or too large, too light or too dark, to have fallen off too early or too late. The cicatrix, too, is declared, if little, to prove that the local affection has been trifling and imperfect; and, if large, it indicates some other diseased action. If the cicatrix is deep or superficial, the same conclusions are drawn. Nay, if it is of the very exact dimensions, still it is too much indented or serrated to allow the process to have been complete. Here I may surely be allowed to enquire, what length are their excuses, and the good humour and indulgence of the public, to be carried? Nothing but the most desperate and headstrong zeal can demand credit for such a tissue of absurdity; and I conceive it perfectly sufficient to enumerate them, in order to produce their refutation and expose their folly.

"The next reason given for still placing confidence in the efficacy of the vaccine inoculation, is 'that a very large proportion is still found to resist the influence of the small-pox contagion.'

"Those gentlemen who have stated this as a sufficient argument for persevering in the vaccine inoculation, do not even hint at what they mean by a very large proportion; but, when we examine the different results which experience now affords, we shall find the most decisive evidence for rejecting any hopes which may be entertained from this quarter."—

"I cannot close this part of our subject without noticing a fact which has been triumphantly (but, in my opinion, very injudiciously and imprudently) employed by the supporters of the vaccine inoculation, for recommending this practice to public confidence: —I mean, that cases of failure have been much more numerous amongst the lower than the higher classes of society. Surely, it is madness itself to contend we have been so base and wicked as to have vaccinated the rich perfectly, and the poor imperfectly; and, if this were the fact, the utility of our vaccine establishments must be more than questionable, as their practice is almost exclusively confined to the lower orders of the community. But this fact can be satisfactorily explained, and is, upon a little reflection, quite obvious. It is well known the higher orders of society uniformly availed themselves either of inoculation or vaccination, and, as they all attend schools, and even in their amusements are still amongst themselves, it is evident small-pox contagion cannot reach them so readily; but, wherever they have been placed in the same circumstances, the same result has uniformly followed; and now these orders of society are taught, from experience, their tenderest concerns have been no better protected than their neighbours. Indeed, this fact, far from being employed as a bait and recommendation, ought, on the contrary, to be considered as the strongest argument against the practice. I am afraid those very individuals, who flatter themselves with this exception, and reject with disdain, and even horror, every attempt to expose the imperfection of the vaccine inoculation, will ultimately prove the only sufferers; as they may avoid the influence of the small-pox contagion while the power of vaccination is yet capable of mitigating the variolous influence, and may be afterwards infected with small-pox, when all the influence of the vaccine disease is exhausted, and left completely at the mercy of the epidemic."—

"We come now to a reason which is made use of by the determined supporters of the vaccine inoculation, which, to my apprehension, is the most singular deviation from the result of general experience to be met with in the whole history of medicine:—I mean, where they allege 'that the security imparted either by the inoculated or natural small-pox is not greater than what is obtained from the vaccine;' or, in other words, 'that small-pox are as liable to occur twice in the same person, as where small-pox are found to occur after cow-pock.' Indeed, this reason appears to me so ridiculous and destitute of all truth, as not to deserve the smallest attention. But, besides what we have formerly noticed, which may be considered as a sufficient answer to so strange an aberration from all experience, we shall also observe, the experience of a thousand years of the natural, and nearly a century of the inoculated, small-pox, have distinctly proved that it may be adopted as a determined general fact,—whoever has once passed through the small-pox in a satisfactory manner will not again be subjected to that disease."—

"We come now to a very curious, and, I rather think, somewhat whimsical, attempt to defend the Jennerian discovery:—I allude to an opinion lately suggested by some medical gentlemen, 'that variola, varicella, and modified small-pox after cow-pock, arise from one and the same contagion;' or, according to my apprehension, 'are one and the same disease.'

"It is really truly surprising that this vaccine inoculation, which was once so simple, so uniform, and so satisfactory, should now become such a puzzle as nearly to overturn the most established principles in the whole history of medicine, and that every well-known fact must bend merely in compliment to those who have staked their existence upon the success of vaccination. This suggestion seems to me devoid of all support from experience and analogy; and, if these gentlemen are to be allowed to change their ground in this very extraordinary manner, it will certainly be a Herculean task to compel them to surrender.

"I can assure you, from an experience of thirty years' full practice, that I have frequently seen varicella prevailing as an epidemic, and not a case of small-pox known in the town or whole neighbourhood: that this was the case during the last winter and spring, and not a case of small-pox existed in Musselburgh. I have also uniformly observed, that varicella goes through the children of the family, with all its characters, in the same manner as variola or rubeola would do; and, both before and since the introduction of vaccination, it has regularly prevailed in the families where the children had all been either previously inoculated or vaccinated. The varicella has been also equally prevalent since the introduction of vaccination, and has attacked children even a month or two after they had undergone the vaccine process. It ought also to have occurred to these gentlemen, that the disease of varicella could not have been less frequent since the introduction of vaccination; for, according to even the advocates of vaccination, cases of varicella have not only been more numerous, but also have been stated to have become fatal since the introduction of the Jennerian practice.* But I apprehend there is not the smallest reason for torturing the proof upon this occasion, as the experience of every medical practitioner must declare, that none but the vaccinated children have been subjected to the influence of small-pox, while all the rest of the family escaped."—

"I have now nearly got through the different reasons urged by the zealous supporters of vaccination, for our persevering to place our confidence in the vaccine inoculation; but, before concluding, I must also notice the very improper defence which is attempted to be obtained by dragging forward the experience of other countries. It surely cannot be fairly contended, that the country in which the disease has had its origin, and by the exertions of which it has been extended over a great part of the globe, can possibly be deficient in that skill and attention necessary for tracing its effects and consequences; and that, after having introduced the disease, we must surrender up our qualifications for farther observation to the medical practitioners of other countries, and only in future be directed by them. The doctrine is so monstrous and absurd as to require no further comment. On the contrary, I apprehend, a heavy responsibility rests upon the medical profession of this kingdom; and it is our peculiar duty to watch over the practice with the most anxious and disinterested concern, that we may not only guard our own, but the security of those whom we have encouraged to place their confidence in the Jennerian discovery, and not allow ourselves to be detected and exposed by those who, in this disease at least, we are entitled to take precedence of; for it is particularly to be remembered, that the vaccine practice is exactly following the same course it has done in this country, and cases of failure are rapidly accumulating in every situation."

---

* "Which last cases, I have no doubt, were all instances of small-pox after cow-pock."

### *Hospital for the Small-Pox, for Inoculation, and for Vaccination, at Pancras.*

An Account of the Number of Deaths occasioned by the Casual Small-Pox, extracted from the register for twenty years before the practice of Vaccination, and also for twenty years since; also the Number of Deaths as reported by the Parish Clerks of London, &c. copied from their general Bills of all Christenings and Burials for the same periods:—

| *Before Vaccination.* | | | *Since Vaccination.* | | |
|---|---|---|---|---|---|
| A.D. | Hosp. Reg. | Par. Reg. | A.D. | Hosp. Reg. | Par. Reg. |
| 1779 to 1798 | 1867 | 36,189 | 1799 to 1818 | 814 | 22,480 |

Decreased in deaths since the practice of vaccination was introduced—at the Hospital, 1053; in the Parishes, 13,709.

Vaccination was introduced into practice at the Hospital for Inoculation, by Dr. Wm. Woodville, with the disease taken from the cows belonging to Thomas Harrison, esq. of Gray's Inn-road, on the 19th January, 1799. Six patients were vaccinated by the Doctor, in the presence of Sir J. Banks, bart. Sir W. Watson, Drs. Garthshore, George Pearson, Robert Willan, and several other medical gentlemen. The number vaccinated from that date to the 1st of January, 1819, amounted to 43,394 at this Hospital.

J. C. WACHSEL, *Resident Surgeon.*

---

### BILLS OF MORTALITY.

By the Company of Parish Clerks of London, for the Year 1818.

### *Diseases and Casualties for the Year* 1818.

| DISEASES. | |
|---|---|
| Abscess | 103 |
| Aged | 1923 |
| Ague | 1 |
| Apoplexy and Suddenly | 512 |
| Asthma | 859 |
| Cancer | 97 |
| Canker | 1 |
| Chicken-pox | 2 |
| Childbed | 221 |
| Consumption | 4242 |
| Convulsions | 3205 |
| Cough and Hooping Cough | 839 |
| Croup | 113 |
| Dropsy | 709 |
| Dysentery | 16 |
| Fevers of all kinds | 1170 |
| Fistula | 9 |
| Gout | 58 |
| Gravel, Stone, Strangury | 17 |
| Hæmorrhage | 43 |
| Inflammation | 1203 |
| Jaundice | 91 |
| Jaw Locked | 1 |
| Liver complaint | 72 |
| Lunacy | 228 |
| Measles | 728 |
| Miscarriage | 2 |
| Mortification | 368 |
| Palpitation of the Heart | 7 |
| Palsy | 187 |
| Pleurisy | 15 |
| Rheumatism | 13 |
| Rupture | 38 |
| Scrofula | 12 |
| Small-pox | 421 |
| Sore Throat | 11 |
| Spasm | 78 |
| St Anthony's Fire | 8 |
| Still-born | 654 |
| Teething | 445 |
| Thrush | 107 |
| Venereal | 19 |
| Water in the Chest | 101 |
| Water on the Brain | 406 |
| Worms | 6 |

| CASUALTIES. | |
|---|---|
| Broken Limbs | 1 |
| Burnt | 33 |
| Drowned | 117 |
| Excessive Drinking | 5 |
| Executed | 11 |
| Found Dead | 14 |
| Fractured | 1 |
| Frighted | 3 |
| Killed by Falls & several other Accidents | 92 |
| Killed by Fighting | 1 |
| Murdered | 2 |
| Poisoned | 6 |
| Scalded | 8 |
| Starved | 1 |
| Strangled | 1 |
| Suffocated | 8 |
| Suicides | 40 |
| Total | 344 |

| | | | |
|---|---|---|---|
| Christened | Males 12,530 | Females 11,703 | In all 24,233 |
| Buried . | Males 9,883 | Females 9,923 | In all 19,705 |

Whereof have died,

| | | | |
|---|---|---|---|
| Under Two Years of Age | 5381 | Sixty and Seventy | 1585 |
| Between Two and Five | 1815 | Seventy and Eighty | 1271 |
| Five and Ten | 808 | Eighty and Ninety | 722 |
| Ten and Twenty | 703 | Ninety and a Hundred | 175 |
| Twenty and Thirty | 1453 | A Hundred | 1 |
| Thirty and Forty | 1884 | A Hundred and One | 1 |
| Forty and Fifty | 2040 | A Hundred and Three | 1 |
| Fifty and Sixty | 1864 | A Hundred and Five | 1 |

Decreased in the Burials this Year 263.

There have been Executed in the City of London and County of Surrey 24; of which Number 11 only have been reported to be buried within the Bills of Mortality.

### REPORT OF DISEASES.

THERE has been no decided variation in the general character of the prevalent fever from that which it possessed during the preceding month. This subject will, therefore, not detain us on the present occasion.

Inflammation of the mucous membrane of the respiratory organs continues to occur with increased frequency. Many cases have been witnessed, especially in elderly persons, where the excitation has been transferred to the serous membranes of the cavity of the chest, giving rise to hydrothórax. The former affection has subsided, and, after a few days, or sometimes several weeks, the symptoms of the latter malady have appeared. Continued experience increases our confidence in the opinion that hydrothorax is, in by far the greater proportion of cases, the consequence of irritation, or inflammation, of the serous membranes of the pectoral cavity. A degree of irritation, or of inflammation, may exist in membranous parts, without being attended with pain, which will induce increased effusion from the exhalants; and thence it is often not detected until the latter effects have ensued. It is in these cases that digitalis has been so beneficial as to lead to the general, and almost indiscriminate, use of that remedy in this affection. We have, in many instances, joined with it local or general bleeding, with the most fortunate results. Repeated blistering, or (what is equally efficacious and much less disagreeable) the production of a pustular rash on the chest by tartar-emetic ointment, should not be neglected.

Croup has been rather frequent amongst infants; and, as usual, has terminated fatally in a considerable proportion of cases, in consequence of its having been neglected in its early stage. We have lately seen one instance which showed, in a forcible manner, the importance of the advice of Professor Schwilgue,—that, on visiting an infant whose complaint may probably be that under consideration, the medical attendant should wait until he witnesses a fit of coughing; he will then detect the nature of the disease, when the freedom of respiration during the intervals might lead him to consider the complaint of but little importance. In the case to which we have alluded, the medical practitioner was deceived from the cause above mentioned, until the disease had assumed its most alarming form. The appropriate mode for the treatment of this serious malady is, we believe, generally employed with sufficient assiduity; yet we would endeavour to impress on the mind of the young practitioner the importance attached to exhibition of an active dose of calomel and jalap immediately after blood-letting; and that in this, as in all cases where the prompt and decisive effects of antimony are required, tartar emetic should be used in preference to the antimonial or James's powder; and that the doses of this medicine should be repeated at least once in an hour.*

Hæmoptoë is somewhat prevalent, and in a few cases has terminated in pneumonia of a serious character.

Dysenteric affections have been rather frequent. These are, however, but trifling in comparison with the fatal malady, similarly denominated, that desolates our armies in moist and hot climates. In enquiring into their cause, the *διὰ τὸν τῆς κοιλίης σκληρότητα*, so incessantly uttered by Hippocrates, immediately and almost constantly presents itself to the civic practitioner; especially in England, where animal food constitutes so large a proportion of the aliment of persons of all ranks.

This is the season for the fatal termination of pulmonary consumption;

---

* We are particularly anxious to direct the attention of the younger part of our readers to this powerful and valuable remedy. Those who may not be already acquainted with it will derive much useful information from the work of Dr. Balfour, which we noticed in a late Number of our Journal.

and it is that also in which the victims of that malady are most disposed to indulge hopes of the restoration of health. Many causes contribute to produce this delusion. The patients have passed through the chief part of the winter; they feel relieved from much distress; their respiration has become more easy; nothing but cough and weakness, they say, remain. But the influence of winter has produced its irremediable effects: by hurrying on the inflammatory state of the malady, it has conducted it to its final stage. When suppuration is fully established, the difficulty of breathing and cough are often considerably mitigated; but these circumstances will not delude the accurate and experienced observer.

### METEOROLOGICAL JOURNAL,

**By Messrs. William Harris and Co. 50, Holborn, London.**

***From the 20th January to the 19th February, 1819, inclusive.***

| Day of Month. | Moon. | Rain gauge | Therm. | | | Barom. | | De Luc's Hygrom. Dry | Damp | | Wind. | | Atmospheric Variation. | | |
|---|---|---|---|---|---|---|---|---|---|---|---|---|---|---|---|
| Jan. | | | | | | | | | | | | | | | |
| 20 | | | 39 | 41 | 37 | 29·67 | 29·48 | | 11 | 11 | SSW | S | Fine | | |
| 21 | | | 39 | 43 | 39 | 29·50 | 29·58 | | 12 | 12 | S | SW | Fine | | |
| 22 | | | 41 | 45 | 38 | 29·70 | 20·48 | | 12 | 12 | SSW | SW | Fine | Rain | Fine |
| 23 | | | 41 | 47 | 40 | 29·65 | 29·79 | | 13 | 12 | SSW | S | Fine | Clo. | |
| 24 | | | 44 | 49 | 40 | 29·62 | 29·55 | | 10 | 11 | SE | SE | Fine | | Rain |
| 25 | | | 43 | 46 | 40 | 29·44 | 29·42 | | 11 | 13 | SSE | SSW | Clo. | Rain | Fine |
| 26 | ● | | 43 | 45 | 42 | 29·48 | 29·52 | | 11 | 11 | S | ESE | Clo. | | |
| 27 | | | 45 | 47 | 39 | 29·41 | 29·40 | | 11 | 12 | SW | S | Clo. | Rain | Clo. |
| 28 | | | 42 | 47 | 41 | 29·43 | 29·55 | | 11 | 11 | S | S | Clo. | | |
| 29 | | | 44 | 50 | 40 | 29·49 | 29·46 | | 11 | 10 | SE | ENE | Fine | | Clo. |
| 30 | | | 44 | 48 | 39 | 29·39 | 29 42 | | 12 | 11 | ENE | N | Rain | | Clo. |
| 31 | | | 41 | 46 | 34 | 29·51 | 29·65 | | 10 | 10 | N | NNW | Clo. | | |
| Feb. | | | | | | | | | | | | | | | |
| 1 | | | 34 | 38 | 33 | 29·64 | 29·57 | | 10 | 11 | SE | SW | Fine | | Rain |
| 2 | ☽ | | 35 | 38 | 30 | 29·61 | 29·78 | | 9 | 10 | NNW | W | Snow | Fine | |
| 3 | | | 33 | 37 | 38 | 29·58 | 29 52 | | 10 | 10 | N | W | Fine | Clo. | Rain |
| 4 | | | 41 | 46 | 39 | 29·70 | 29·84 | | 10 | 11 | WNW | WSW | Fine | Rain | Clo. |
| 5 | | | 42 | 45 | 40 | 29·72 | 29·65 | | 11 | 12 | SW | SW | Rain | | |
| 6 | | | 40 | 43 | 42 | 29·67 | 29·65 | | 11 | 12 | SW | W | Fine | | Rain |
| 7 | | | 45 | 47 | 38 | 29·65 | 29·60 | | 11 | 11 | SW | SW | Fine | Rain | Fine |
| 8 | | | 41 | 46 | 44 | 29·89 | 30·02 | | 11 | 12 | WSW | SW | Fine | | Clo. |
| 9 | | | 46 | 49 | 41 | 29·94 | 29·80 | | 14 | 14 | SW | SSW | Rain | | |
| 10 | ○ | | 43 | 47 | 40 | 29;88 | 30·07 | | 11 | 11 | SW | SW | Fine | | |
| 11 | | | 43 | 47 | 39 | 29·98 | 29·92 | | 10 | 10 | SW | W | Fine | Clo. | |
| 12 | | | 40 | 46 | 40 | 29·88 | 29·62 | | 11 | 13 | WSW | W | Fine | Rain | |
| 13 | | | 42 | 46 | 38 | 29·85 | 29·91 | | 12 | 15 | W | NW | Rain | | Clo. |
| 14 | | | 40 | 43 | 37 | 30·00 | 30·11 | | 15 | 14 | NW | W | Fine | | |
| 15 | | | 40 | 46 | 35 | 30·00 | 29·70 | | 12 | 12 | W | W | Clo. | | Rain |
| 16 | | | 40 | 46 | 36 | 29·61 | 29·44 | | 12 | 15 | SW | SSW | Clo. | | Rain |
| 17 | ☾ | | 39 | 46 | 35 | 29·50 | 29·54 | | 14 | 12 | SSW | S | Rain | Clo. | |
| 18 | | | 41 | 47 | 38 | 29·64 | 29·55 | | 13 | 15 | W | S | Clo. | | Rain |
| 19 | | | 44 | 49 | 40 | 29·61 | 29·73 | | 13 | 12 | SW | W | Rain | Fine | |

The probable (the gauge being not quite perfect) quantity of rain fallen in January is 2 inch and 11-100ths.

